# PRO: home sleep studies in children—are we there yet?

**DOI:** 10.1093/sleep/zsad040

**Published:** 2023-02-24

**Authors:** Hui-Leng Tan, Athanasios G Kaditis

**Affiliations:** Department of Pediatric Respiratory Medicine, Royal Brompton Hospital, London, UK; Division of Pediatric Pulmonology, First Department of Pediatrics, National and Kapodistrian University of Athens School of Medicine and Agia Sofia Children’s Hospital, Athens, Greece; Division of Pediatric Pulmonology and Pediatric Sleep Center, Department of Child Health, University of Missouri School of Medicine and MUHC Children’s Hospital, Columbia, MO, USA

## Argument

The following argument was prepared in response to the question without the knowledge of the contents of the opposing argument.

Whilst in-laboratory polysomnography (PSG) is the current gold standard for the diagnosis of pediatric obstructive sleep apnea syndrome (OSAS), it is expensive, labor-intensive, and more importantly, not readily accessible to all children globally. Some children do not tolerate the numerous sensors required and some may not sleep as well in the unfamiliar environment of a sleep laboratory. Adult sleep medicine physicians have embraced the clinical use of home sleep apnea testing, publishing clear guidance on its use [1]. In contrast, in 2017 an American Academy of Sleep Medicine Task Force of experts recommended against using a home sleep apnea test (HSAT) for the diagnosis of OSAS in children [2]. In this Pro Editorial, we will argue that this position needs to be reconsidered in light of the significant new research published on HSATs in the subsequent half-decade since the position paper was published. That work is further reinforced by the seismic shift in the landscape of how sleep medicine is delivered, engendered by the impact of the SARS-CoV-2 global pandemic.

The usual indications for a sleep study include clinical symptoms, signs and endoscopy or imaging findings consistent with OSAS, central sleep apnea, sleep hypoventilation, or hypoxemia, and their combinations (sleep-disordered breathing-[SDB]) [3, 4]. SDB may affect otherwise healthy children or patients with comorbidities like neuromuscular disorders, craniofacial abnormalities, Prader-Willi syndrome, or Trisomy 21.

Most commercially available HSAT devices incorporate a minimum of four channels, without electroencephalogram (EEG) recording (level III) or 1–2 cardiorespiratory channels such as oximetry, pulse rate, airflow, or respiratory rate (level IV) [5]. Our proposition is that Level III or IV devices can be utilized at the patient’s home for diagnosing OSAS in childhood. Emphasis will be given to diagnosis of SDB in otherwise healthy children but available studies in children with comorbidities will also be discussed. We suggest that: (1) Level III or IV devices can reliably detect moderate-to-severe OSAS (apnea–hypopnea index-AHI >5 episodes/h) so that necessary treatment interventions will not be inappropriately omitted, (2) mild OSAS (apnea-hypopnea index [AHI] 1–5 episodes/h) without SDB-associated morbidity will not be misdiagnosed as disease of moderate severity resulting in unnecessary treatment interventions, and (3) Level III and IV devices can reliably detect severe OSAS so that major respiratory complications post-adenotonsillectomy are anticipated and appropriate postoperative monitoring is planned. We will also address arguments against the use of HSATs in the pediatric population that were discussed in the 2017 American Academy of Sleep Medicine position paper (Table 1) [2].

**Table 1:** Limitations of HSATs for the diagnosis of OSAS as reported in the American Academy of Sleep Medicine position paper [15] and respective pro arguments in view of recently published evidence

1. *HSATs may be technically feasible in the pediatric population if electrodes are placed by trained professionals but likelihood of success may be significantly reduced if sensors are placed by caregivers.* *An approximately 90% rate of technically acceptable recordings has been reported when HSATs are set up by caregivers at the child’s home.*
2. *There are few published studies comparing the validity of HSATs against polysomnography (gold standard) in children and most were performed in the sleep laboratory.* Recent validation studies have shown that unattended sleep studies using Level III devices at home can predict moderate-to-severe OSAS diagnosed by polysomnography with a sensitivity of 62%–100% and specificity of 93%–100%.
3. *Scoring of hypopneas accompanied by arousals but not desaturation is not possible since Type III devices for HSATs do not include EEG recordings; data on total sleep time are not provided and total recording time is included instead in the denominator of respiratory indices leading to underestimation of the apnea–hypopnea index and underdiagnosis of OSAS and its severity.* Total sleep time can be approximated if a record of study events (wakefulness, sleep, feeding, snoring, apnea) is prepared by motivated caregivers. Misdiagnosing moderate-to-severe OSAS as mild disease due to AHI underestimation will not modify treatment recommendations in patients with OSAS-associated morbidity, factors predicting OSAS persistence, or complex disorders.
4. *Devices for HSATs do not include end-tidal or transcutaneous CO2 monitoring and detection of hypoventilation is not possible.* End-tidal or transcutaneous carbon dioxide partial tension monitoring is unnecessary in otherwise healthy children with suspected OSAS, but it may be added to a HSAT to avoid missing the diagnosis of hypoventilation when patients with comorbidities are evaluated.
5. *There are limited data on the feasibility or validity of HSATs use in children with comorbid medical conditions.* HSATs are feasible in children with comorbid disorders like Down syndrome and neuromuscular disorders with the frequency of obtaining interpretable results ranging from 55% to 90% most likely depending on the device used and the instructions provided to the caregivers for setting up the equipment. There are limited data validating HSATs against in-laboratory polysomnography in children with special needs.
6. *There are no feasibility or validity data for the application of HSATs in children younger than 2 years.* It is feasible to use type III devices for HSATs at home in young children and obtain technically acceptable recordings provided that a trained professional places the sensors. However, there are no validation studies comparing the method to in-laboratory polysomnography.

### What are the arguments against performing HSATs?

A number of arguments against the use of HSATs have been presented: (1) at the time of literature review by the American Academy of Sleep Medicine Task Force, there was a limited number of feasibility and validity studies on the use of HSATs and most of them were completed in the sleep laboratory and not at patients’ homes, (2) available devices do not include sensors for monitoring arousals and carbon dioxide partial pressure leading to underestimation of SDB severity, (3) total sleep time cannot be calculated and total recording time is used instead in the denominator of the AHI, and (4) there are limited studies comparing HSATs to PSG and especially in young children and those with medical comorbidities [1].

### HSATs are technically feasible in children without comorbidities

In a large, multicenter study involving 201 children, Marcus et al. have shown that comprehensive, unattended, home PSG is feasible, provides technically adequate recordings (91% of cases), and is well tolerated in school-aged children when sensors are placed by a sleep technologist [6]. A few studies published prior to 2017 have also demonstrated that HSATs involving level III devices are technically feasible provided that the equipment is set up by a trained professional at the child’s home, while the likelihood of obtaining interpretable recordings may decrease significantly if caregivers are assigned the responsibility to place sensors [2]. Most common technical problems that adversely affect the quality of the HSATs are poor SpO_2_, nasal airflow, or thoracoabdominal movements tracings [7, 8].

However, studies published after 2017 have demonstrated that technically acceptable recordings are obtained in approximately 90% of cases where caregivers placed the sensors at home [7, 9, 10]. More specifically, Scalzitti et al. have reported a 94% rate of technically acceptable recordings when a type III device was set up in the sleep laboratory versus 89% when sensors are placed at home by caregivers [7]. Success rate could probably improve further if remote monitoring of HSAT recordings via a tablet device by the on-call sleep technologist is available, who can then provide families with overnight support [11].

### Validation studies indicate that HSATs based on Level III devices can predict moderate-to-severe OSAS diagnosed by PSG in children without comorbidities

If type III devices are set up, attended to, and scored manually by trained professionals, they can provide reliable results in otherwise healthy children suspected of OSAS despite their technical limitations [12–14]. HSATs are characterized by satisfactory specificity (83.3%–95.8%) and negative predictive value (86.8%–95.2%) along with modest-to-satisfactory sensitivity (68.2%–96.1%) and positive predictive value (67%–88.2%) for detecting moderate-to-severe OSAS.

In addition, increasing evidence suggests that unattended sleep studies in children without comorbidities using Level III devices at home can predict moderate-to-severe OSAS diagnosed by PSG with sensitivity of 62%–100% and specificity of 93%–100% [15]. A recent meta-analysis included in the British Thoracic Society (BTS) Guideline for diagnosing and monitoring pediatric SDB has shown that HSATs have sensitivity of 85% (95% CI 55%, 96%) and specificity of 71% (95% CI 39%, 90%) for predicting AHI >1–5 episodes/h among children without or with comorbidities [15]. Although these are not perfect values reflecting a highly accurate test, HSATs may be more cost-effective without major health repercussions than in-laboratory PSG. More compellingly, making HSATs available to patients could mean that the numerous children with moderate-to-severe OSAS who currently have no access to a sleep laboratory can be diagnosed and treated.

### Inability to score arousals, calculate total sleep time, and identify hypoventilation when using level III devices at home does not always prevent appropriate treatment recommendations in otherwise healthy children

AHI calculated from Level III device recordings may be underestimated due to (1) under-recognition of hypopneas not accompanied by intermittent hypoxemia since related arousals cannot be scored, and (2) use of total recording time in the denominator of respiratory indices because EEG and electromyography (EMG) are not recorded and total sleep time cannot be calculated. This underestimation decreases sensitivity and negative predictive value and increases specificity and positive predictive value for detecting moderate-to-severe OSAS. Total sleep time can be approximated if a record of study events (wakefulness, sleep, feeding, snoring, and apnea) is prepared by motivated caregivers.

In contrast, and depending on the specific level III device applied at home, AHI may be overestimated because (1) “pseudo-events” secondary to either reduced amplitude of the nasal airflow channel resulting from mouth breathing and/or artifactually reduced flow post-arousal can be recorded [9, 16], and (2) respiratory events can be scored during wakefulness [12]. Overestimation of the AHI has been reported less frequently than underestimation of the AHI.

Despite general agreement that a child with moderate-to-severe OSAS (AHI >5 episodes/h) should receive treatment, there are many other examples that treatment interventions may also be beneficial to patients irrespective of the AHI. Such patients are those with mild OSAS and history of associated morbidity from the cardiovascular or central nervous systems (e.g. hypertension, poor school performance, or lack of attention), enuresis, somatic growth delay, or failure, decreased quality of life or presence of factors predicting OSAS persistence (male sex, obesity, tonsillar hypertrophy) [3]. Moreover, children with mild SDB and complex disorders predisposing to upper airway obstruction like major craniofacial abnormalities or neuromuscular disorders also need treatment to avoid OSAS deterioration and development of pulmonary hypertension [3]. Misdiagnosing moderate-to-severe OSAS as mild disease due to AHI underestimation in such cases will not modify treatment recommendations. Overestimation of AHI by HSAT is more problematic when a child with mild OSAS and without SDB-associated morbidity, that may resolve spontaneously, is diagnosed with moderate-to-severe disease and is inappropriately offered adenotonsillectomy or noninvasive ventilation. It is thus reasonable to suggest that, when the HSAT results do not match the clinical presentation, an in-laboratory PSG is performed prior to making treatment recommendations.

Although there are two recent examples of level III devices that can monitor end-tidal or transcutaneous carbon dioxide partial pressure by interfacing with capnometers, capnography is not an option in the majority of home sleep study devices [10, 17]. Nevertheless, it has been shown that overnight carbon dioxide monitoring is not necessary for the diagnosis of OSAS in otherwise healthy children, but is crucial in children with comorbidities such as Prader-Willi or Down syndrome, neuromuscular disease, or craniofacial abnormalities who may have nocturnal hypoventilation [18]. Therefore, end-tidal or transcutaneous carbon dioxide partial tension monitoring may be added to a HSAT to avoid missing the diagnosis of hypoventilation in patients with comorbidities. The success rate of oxycapnography at home in one feasibility study performed during the coronavirus disease 2019 pandemic lockdown was 83% [11].

### Emerging evidence demonstrates the feasibility of HSATs in children with comorbid medical conditions and those aged 1–2 years

The BTS Guideline for diagnosing and monitoring pediatric SDB suggests that sleep studies at home using level III devices can also be considered for children with comorbidities if the patient and caregiver are deemed appropriate for this type of diagnostic test [15]. Two studies from the UK have explored the feasibility of HSATs set up by caregivers in almost 300 children with Down syndrome or a variety of other disorders like achondroplasia, cerebral palsy, and other neuromuscular diseases [11, 17]. The frequency of obtaining successful recordings (≥4 h of interpretable sleep data) at first attempt ranged from 56% to 74%.

At least two additional recent studies have validated HSATs against in-laboratory PSG in a total of 47 children with Down syndrome or neuromuscular disease utilizing level III devices set up by caregivers [9, 10]. In the study by Ikizoglu et al., 89% of HSATs were technically successful and all participants diagnosed with moderate-to-severe OSAS (AHI >5 episodes/h) by PSG were classified in the same SDB severity category by HSAT [9]. In contrast, Fishman et al reported that HSATs incorrectly classified one-third of participants with moderate-to-severe OSAS as having mild OSAS, while end-tidal carbon dioxide monitoring was successful in only 50% of studies. The investigators suggested that a level III device with capnography is not yet suitable to be implemented in clinical practice as a diagnostic tool for SDB in pediatric neuromuscular disease. In summary, sensors of type III devices can be placed by caregivers at home in children with comorbidities and the quality of recordings is variable, while there are limited data for the validity of the obtained AHI relative to the value calculated by in-laboratory PSG.

HSATs involving type III devices are feasible in 1-year-old infants provided that a trained professional completes equipment setup at the patient’s home. Vezina et al. performed home sleep cardiorespiratory monitoring among 562 infants (mean age 1.1 ± 0.2 years) participating in the Canadian Healthy Infant Longitudinal Development Study to calculate reference values for respiratory parameters during sleep and reported a 91% rate of technically acceptable studies [19].

Data are limited on specific age groups who may be more suitable for HSATs and on the validity of results as compared to PSG. Scalzitti et al. assessed the association between age and technically unsuccessful studies and showed no significant difference in feasibility, but there was a greater margin of error in AHI values relative to PSG in the younger age group (<6 years old) than in the older age group (6–18 years old) [7]. Kingshott et al. offered HSATs to children with Down syndrome some of whom were younger than 2 years, but without comparing results to those of in-laboratory PSG [17]. In summary, it is feasible to use type III devices for HSATs in children aged 1–2 years and to obtain technically acceptable recordings if trained professional places the sensors, but there are no validation studies comparing the method to in-laboratory PSG.

### Level IV devices can be used at home for the diagnosis of moderate-to-severe OSAS

When nocturnal oximetry is applied at home for the diagnosis of OSAS, McGill Oximetry Score (MOS), and the Oxygen Desaturation (≥3%) Index (ODI3) are usually utilized as predictors of OSAS severity. MOS varies from one (normal or inconclusive study) to four (severe hypoxemia) depending on the number of clusters and depth of desaturations. Among children with suspected OSAS and without or with comorbidities, MOS >2 has excellent specificity (100%) and positive predictive value (100%), but fair sensitivity (59%) and satisfactory negative predictive value (78%) for moderate-to-severe OSAS [20].

In a recent study, an ODI3 ≥4.2 episodes/hour had sensitivity of 92.7%, specificity of 78.9%, positive predictive value of 80.9%, and negative predictive value of 91.8% for predicting moderate-to-severe OSAS in otherwise healthy children with snoring [21]. ODI3 emerges as a useful complementary oximetry parameter to MOS. Introduction of commercially available software based on machine learning algorithms for the automatic interpretation of nocturnal oximetry is anticipated to allow the efficient screening of large numbers of children with snoring for OSAS [22].

The Australasian Sleep Association Guidelines for Technical Specifications and Interpretation of Overnight Oximetry for evaluating Pediatric OSAS discourage its use for this purpose in infants. Physiologic central apneas of infancy may be associated with increased frequency of desaturations which could spuriously be considered as corresponding to obstructive events [23].

### Nocturnal oximetry parameters can predict severe OSAS and identify otherwise healthy children at risk of respiratory compromise post-adenotonsillectomy

Parameters of PSG describing episodes of oxygen desaturation of hemoglobin rather than the AHI predict postoperative respiratory complications following adenotonsillectomy, namely admission to the intensive care unit, reintubation, or need for oral or nasal airway, or noninvasive ventilation [24]. Hence, nocturnal oximetry at home is an attractive method to identify those children with symptoms or signs of OSAS who will need close monitoring postoperatively. A common practice in the Canadian public health system is that children with preoperative MOS of three (moderate hypoxemia) stay overnight in the hospital post-adenotonsillectomy, while those with MOS of four (severe hypoxemia) are monitored in the pediatric intensive care unit. In a retrospective cohort of 362 otherwise healthy children who underwent adenotonsillectomy for OSAS, only one child with MOS of four had major respiratory compromise postoperatively requiring reintubation [25].

### HSATs may reduce healthcare disparities and costs and are preferable to in-laboratory sleep studies by caregivers

Communities with limited resources might have lower availability of pediatric sleep laboratories. It is thus likely that OSAS is underdiagnosed in children living in those communities and adenotonsillectomy is offered based on parental reports of OSAS symptoms and physical examination findings [3]. In a retrospective cohort study, among 27 837 children younger than 10 years who had adenotonsillectomy for OSAS, only 12.8% underwent PSG within 18 months before surgery [26]. Of concern, shorter driving time/distance from the sleep center was associated with increased odds of undergoing PSG. Offering HSATs even with devices that have limited technical capabilities may help to reduce health care inequality between regions with poor and adequate resources like rural and urban, respectively. It is worth mentioning that the cost of a HSAT in the US ranges from 200 USD to 450 USD which is considerably lower than the 650 USD to 1250 USD amount reimbursed from insurance providers for in-laboratory PSG.

In addition to HSATs' improved convenience, cost-effectiveness, and accessibility, their utility is further enhanced by their capacity to represent a more typical night’s sleep by avoiding the disturbances in routine, caused by testing in the unfamiliar environment of a sleep laboratory. As Jacob et al. have shown in their study comparing HSATs with in-laboratory PSG, the sleep efficiency may be significantly greater at home than in the sleep laboratory with a mean difference of 5.0% [27]. Certainly, when surveyed, parental perception of the improved comfort and ease of use resulted in the majority rating HSAT as their preferred diagnostic modality [11, 17]. These findings were further reinforced during the coronavirus disease 2019 pandemic lockdown when most sleep laboratories were forced to shut, meeting clinical needs by pivoting to HSATs. Despite the subsequent relaxation of restrictions, many sleep laboratories have opted to continue offering some patients HSATs. This evolution of service delivery models again highlights the perceived real-world advantages of HSATs.

## Conclusions and Future Perspectives

There is no doubt that objective evaluation of the exact pattern and severity of SDB in children facilitates the choice of the right timing and type of treatment intervention(s). Given that at least 10% of the otherwise healthy pediatric population-and even more among children with comorbidities-have symptoms of SDB, it is unrealistic to expect that in-laboratory PSG will be offered to millions of eligible children across the globe. Not offering the opportunity of HSATs, while waiting for devices with ideal technical specifications, will only increase healthcare disparities, especially in geographic areas with poor resources. There is increasing evidence that unattended sleep studies using Level III devices at home in otherwise healthy children older than 2 years with suspected OSAS are technically feasible and can predict moderate-to-severe OSAS diagnosed by PSG with a sensitivity of 62%–100% and specificity of 93%–100%. Feasibility of the study and validity of its results are probably affected by the type of device, the quality of instructions about equipment setup provided to caregivers and their motivation. HSATs are also feasible in children with comorbidities but evidence on validity of their results compared to PSG is limited.

A scheme involving a referral sleep center covering a large geographic area and coordinating HSATs in cooperation with primary care practices, medical equipment companies, and health insurance organizations will likely increase the coverage of eligible children who will benefit from objective sleep testing. Currently, there are at least 17 commercially available Level III or IV devices with published data describing their application in children. Therefore, the referral sleep center could select one or two devices that have been validated against in-laboratory PSG and with which the sleep team is most familiar and confident about the provided results. Establishment of patient registries for recording HSATs results and patient-important outcomes would promote research on health quality gains and reduced costs. In turn, this would potentially encourage manufacturers to further improve the technical specifications of devices for home pediatric sleep studies as well as persuade health insurance organizations to improve their structures, to maximize these benefits. Just waiting for the technically perfect HSAT device gets us nowhere.

## Rebuttal to Opposing Argument

The following was prepared in response to the opposing argument.

According to the American Academy of Sleep Medicine (AASM) recommendations, in-laboratory PSG is the “gold standard” for evaluating SDB severity in children [2, 28]. In the “Con” argument piece, one of the concerns is that widespread adoption of HSATs could potentially increase the risk of substandard care for children with sleep disorders. We believe that if appropriate steps are taken, not only should this not happen, but the reverse will be found with improvements in the quality of care for the millions of children who do not currently have access to objective testing, due to the limited number of pediatric sleep laboratories around the globe.

### “Conducting sleep studies in children requires both a child-centered comprehensive approach and a controlled environment to ensure feasibility, reliability, and validity of results.”

We cannot agree more that specialized training, ongoing education, and exposure to an adequate volume of patients are required to conduct and score pediatric sleep studies. However, this does not necessarily require a full PSG for every child. The reliability of any innovative change of practice requires careful monitoring, and to enhance feasibility, reliability, and validity of HSATs, we would suggest that sensor setup could preferentially be performed by a pediatric sleep technologist or other trained health professional either at the child’s home or alternatively at the sleep laboratory in the early evening prior to the HSAT if possible. Moreover, scoring and interpretation must be completed by qualified pediatric sleep medicine technologists and physicians from specialized sleep centers who have adequate experience in their interpretation.

Since capnography is not available in the majority of devices designed for home use, otherwise healthy children with SDB symptoms and adenotonsillar hypertrophy and/or obesity could be prioritized for HSATs, while in-laboratory, PSG appointment slots are reserved for children with comorbidities such as Prader-Willi or Down syndrome, neuromuscular disease, or craniofacial abnormalities who are at higher risk of nocturnal hypoventilation [18].

Frequency of reliability issues engendered by equipment failures related to poor tolerance or misplacement of sensors can be reduced if remote advice is provided to caregivers during the study by the on-call sleep technologist. Indeed, some commercial devices allow real-time online transfer of the recordings to a PC or tablet in the sleep laboratory for instant remote signal checks and for providing feedback to caregivers.

### “Policies and procedures to monitor and ensure safety in the sleep lab are critically important in the pediatric population. Appropriate triaging of patients is fundamental to successful integration of pediatric sleep diagnostic services.”

Ensuring patient safety during PSG, for example, by providing supplemental oxygen, continuous positive airway pressure, or noninvasive ventilation in order to address the detected severe gas exchange abnormalities is indeed of paramount importance. Nevertheless, safety issues are more common in patients with comorbidities than in otherwise healthy children with reported snoring and apneas. Hence, triaging of otherwise healthy children for HSATs will possibly increase availability of PSG slots in the sleep laboratory to pediatric patients with significant medical, psychiatric, and neurodevelopmental comorbidities, or children with high likelihood of severe SDB. We completely agree that consensus on triaging procedures for in-laboratory PSG versus HSAT is urgently needed.

### “Family-centered care is a mandatory component of pediatric sleep diagnostic services.”

Noise levels at home, caregiver limitations, and household crowding can admittedly affect HSAT findings. These important adverse consequences of an unfavorable family environment on sleep and breathing may be missed when a sleep study is conducted in the standardized conditions of the sleep laboratory. Therefore, the concern that sleep monitoring may be completed in the unfavorable home environment where the child usually lives is not necessarily a limitation of a HSAT, but may even be a strength, as this is the environment within which the child resides, not the sterile environment of a sleep laboratory.

### “Comprehensive clinical care, including follow-up care for pediatric sleep patients undergoing diagnostic procedures, is a necessary component of an integrated sleep medicine program.”

We would like to further emphasize the statement in the “Con” manuscript that all children and adolescents who have symptoms or physical examination findings which are indicative of obstructive SDB should be referred by their primary care provider to a pediatric sleep specialist for further evaluation [3, 4]. A HSAT is intended to accelerate objective testing for assessment of SDB severity and to facilitate treatment recommendations given the shortage of pediatric sleep laboratories, and not to limit access of children to specialized care, but rather the reverse. As it was very accurately stated in the “Con” article, children with symptoms consistent with OSAS have frequently additional sleep disorders which require evaluation and management by a provider with special training in pediatric sleep medicine [29, 30]. Inappropriate ordering and/or interpretation of HSATs and patient follow-up by non-sleep providers will most likely increase healthcare costs and more importantly inappropriate treatment. In fact, we believe that pediatric HSATs should be performed, analyzed, and patients managed only by trained specialists and not by non-sleep providers or adult centers with little experience of pediatric patients.

### “Recommendations regarding conduction of sleep studies in children need to be based on evidence-based quality metrics and not on insurance/financial considerations.”

The vast majority of HSATs currently performed in Europe are by pediatric sleep centers, not adult ones. We are in agreement that any sleep laboratories for adults that opt to offer PSG to children must follow pediatric standards of care and more specifically appropriate staffing ratios, accommodation of accompanying caregivers, as well as PSG scoring and interpretation in accordance with the AASM rules [31]. It is so important to remember that children are not “little adults.”

We also firmly agree that finance should never be the primary driver of pediatric care. However, any healthcare system, insurance or nationally funded, that seeks to be sustainable in the long term, cannot ignore the need to target their resources as economically as possible.

In conclusion, we propose that HSATs can be offered to otherwise healthy children older than 2 years who have been evaluated in a pediatric sleep center for suspected obstructive SDB. If the type III device is set up under the direct or remote guidance of a trained sleep professional and the recording is scored and results are interpreted by pediatric sleep center staff, HSAT can diagnose moderate-to-severe OSAS with satisfactory sensitivity and high specificity. Such an approach will increase the availability of objective testing for SDB for otherwise healthy children and at the same time will release PSG appointment slots for patients with complex disorders.

## Notes

This article is a written debate between the author(s) of this article and the author(s) of the opposing argument.

## References

[1] Rosen IM, et al. Clinical use of a home sleep apnea test: an updated american academy of sleep medicine position statement. J Clin Sleep Med. 2018;14(12):2075–2077. 10.5664/jcsm.7540 30518456 PMC6287732

[2] Kirk V, et al. American academy of sleep medicine position paper for the use of a home sleep apnea test for the diagnosis of OSA in children. J Clin Sleep Med. 2017;13(10):1199–1203. 10.5664/jcsm.6772 28877820 PMC5612636

[3] Kaditis A, et al. Obstructive sleep disordered breathing in 2- to 18-year-old children: diagnosis and management. Eur Respir J. 2016;47(1):69–94. 26541535 10.1183/13993003.00385-2015

[4] Kaditis AG, Alonso Alvarez ML, Boudewyns A, et al. ERS statement on obstructive sleep disordered breathing in 1- to 23-month-old children. Eur Respir J. 2017;50(6): pii: 1700985. 10.1183/13993003.00985-2017 29217599

[5] Ferber R, et al. Portable recording in the assessment of obstructive sleep apnea. ASDA standards of practice. Sleep. 1994;17(4):378–392. 10.1093/sleep/17.4.378 7973323

[6] Marcus CL, et al. Feasibility of comprehensive, unattended ambulatory polysomnography in school-aged children. J Clin Sleep Med. 2014;10(8):913–918. 10.5664/jcsm.3970 25126039 PMC4106947

[7] Scalzitti N, et al. Comparison of home sleep apnea testing versus laboratory polysomnography for the diagnosis of obstructive sleep apnea in children. Int J Pediatr Otorhinolaryngol. 2017;100:44–51. 10.1016/j.ijporl.2017.06.013 28802385

[8] Michelet M, et al. Successful home respiratory polygraphy to investigate sleep-disordered breathing in children. Sleep Med. 2020;68:146–152. 10.1016/j.sleep.2019.11.1264 32036287

[9] Ikizoglu NB, et al. Are home sleep studies useful in diagnosing obstructive sleep apnea in children with down syndrome? Pediatr Pulmonol. 2019;54(10):1541–1546. 10.1002/ppul.24440 31290291

[10] Fishman H, et al. The accuracy of an ambulatory level iii sleep study compared to a level i sleep study for the diagnosis of sleep-disordered breathing in children with neuromuscular disease. J Clin Sleep Med. 2018;14(12):2013–2020. 10.5664/jcsm.7526 30518444 PMC6287726

[11] Jones S, et al. Feasibility and parental perception of home sleep studies during COVID-19: a tertiary sleep centre experience. Arch Dis Child. 2022;107(2):189–191. 10.1136/archdischild-2021-322184 34551900

[12] Masoud AI, et al. Validation of the medibyte portable monitor for the diagnosis of sleep apnea in pediatric patients. J Clin Sleep Med. 2019;15(5):733–742. 10.5664/jcsm.7764 31053204 PMC6510685

[13] Alonso-Alvarez ML, et al. Reliability of home respiratory polygraphy for the diagnosis of sleep apnea in children. Chest. 2015;147(4):1020–1028. 10.1378/chest.14-1959. 25539419 PMC4388115

[14] Lesser DJ, et al. The utility of a portable recording device for screening of obstructive sleep apnea in obese adolescents. J Clin Sleep Med. 2012;8(3):271–277. 10.5664/jcsm.1912 22701384 PMC3365085

[15] Evans H, et al. Guideline for diagnosing and monitoring paediatric sleep disordered breathing. Thorax. 2022. In press. 10.1136/thorax-2022-218938 37295792

[16] Massicotte C, et al. The utility of a portable sleep monitor to diagnose sleep-disordered breathing in a pediatric population. Can Respir J. 2014;21(1):31–35. 10.1155/2014/271061 24083303 PMC3938237

[17] Kingshott RN, et al. Cardiorespiratory sleep studies at home: experience in research and clinical cohorts. Arch Dis Child. 2019;104(5):476–481. 10.1136/archdischild-2018-315676 30455364

[18] Trucco F, et al. Reducing the need for carbon dioxide monitoring in the investigation of paediatric sleep disordered breathing. Eur Respir J. 2018;52(4):1801290. 10.1183/13993003.01290-2018 30115615

[19] Vezina K, et al. Cardiorespiratory monitoring data during sleep in healthy canadian infants. Ann Am Thorac Soc. 2020;17(10):1238–1246. 10.1513/AnnalsATS.201909-703OC. 32678717

[20] Jonas C, et al. Comparison of nocturnal pulse oximetry with polysomnography in children with sleep disordered breathing. Sleep Breath. 2020;24(2):703–707. 10.1007/s11325-019-01861-z 31104209

[21] Polytarchou A, et al. Nocturnal oximetry parameters as predictors of sleep apnea severity in resource-limited settings. J Sleep Res. 2023;32(1):e13638. 10.1111/jsr.13638. Epub 2022 May 27. 35624085

[22] Gutierrez-Tobal GC, et al. Reliability of machine learning to diagnose pediatric obstructive sleep apnea: systematic review and meta-analysis. Pediatr Pulmonol. 2022;57(8):1931–1943. 10.1002/ppul.25423 33856128 PMC11556234

[23] Twiss J, et al. Overnight oximetry for evaluating paediatric obstructive sleep apnoea: Technical specifications and interpretation guidelines. J Paediatr Child Health. 2019;55(10):12791279.–127911279. 10.1111/jpc.14586 31629377

[24] Molero-Ramirez H, et al. Polysomnography parameters assessing gas exchange best predict postoperative respiratory complications following adenotonsillectomy in children with severe OSA. J Clin Sleep Med. 2019;15(9):1251–1259. 10.5664/jcsm.7914 31538596 PMC6760392

[25] Horwood L, et al. Testing for pediatric obstructive sleep apnea when health care resources are rationed. JAMA Otolaryngol Head Neck Surg. 2014;140(7):616–623. 10.1001/jamaoto.2014.778 24851855

[26] Radhakrishnan D, et al. Geographic disparities in performance of pediatric polysomnography to diagnose obstructive sleep apnea in a universal access health care system. Int J Pediatr Otorhinolaryngol. 2021;147:110803. 10.1016/j.ijporl.2021.110803 34198156

[27] Jacob SV, et al. Home testing for pediatric obstructive sleep apnea syndrome secondary to adenotonsillar hypertrophy. Pediatr Pulmonol. 1995;20(4):241–252. 10.1002/ppul.1950200407 8606854

[28] Aurora RN, et al. Practice parameters for the respiratory indications for polysomnography in children. Sleep. 2011;34(3):379–388. 10.1093/sleep/34.3.379 21359087 PMC3041715

[29] Owens JA, et al. Effect of weight, sleep duration, and comorbid sleep disorders on behavioral outcomes in children with sleep-disordered breathing. Arch Pediatr Adolesc Med. 2008;162(4):313–321. 10.1001/archpedi.162.4.313 18391139

[30] Meltzer LJ, et al. Prevalence of diagnosed sleep disorders in pediatric primary care practices. Pediatrics. 2010;125(6):e1410–e1418. 10.1542/peds.2009-2725 20457689 PMC3089951

[31] Berry RB, Quan SF, Abreu AR, et al. for the American Academy of Sleep Medicine. The AASM Manual for the Scoring of Sleep and Associated Events: Rules, Terminology and Technical Specifications. Darien, IL: American Academy of Sleep Medicine; 2020.

